# Takotsubo Cardiomyopathy in the Setting of Tension Pneumothorax

**DOI:** 10.1155/2015/536931

**Published:** 2015-08-23

**Authors:** Michael Gale, Pablo Loarte, Brooks Mirrer, Thierry Mallet, Louis Salciccioli, Alison Petrie, Ronny Cohen

**Affiliations:** ^1^Division of Cardiology, Department of Medicine, Woodhull Medical Center, 760 Broadway, Suite 3B320, Brooklyn, NY 11206, USA; ^2^Department of Medicine, Yale-New Haven Hospital, 20 York Street, CB2041, New Haven, CT 06510, USA; ^3^NYU School of Medicine, 550 First Avenue, New York, NY 10016, USA; ^4^Division of Cardiology, Department of Medicine, SUNY Downstate Medical Center, 450 Clarkson Avenue, Brooklyn, NY 11203, USA; ^5^Saint George's University School of Medicine, 3500 Sunrise Highway, Great River, NY 11739, USA

## Abstract

*Background*. Takotsubo cardiomyopathy is defined as a transient left ventricular dysfunction, usually accompanied by electrocardiographic changes. The literature documents only two other cases of Takotsubo cardiomyopathy in the latter setting. *Methods*. A 78-year-old female presented to the ED with severe shortness of breath, hypertension, and tachycardia. On physical exam, heart sounds (S1 and S2) were regular and wheezing was noticed bilaterally. We found laboratory results with a WBC of 20.0 (103/*μ*L), troponin of 16.52 ng/mL, CK-mb of 70.6%, and BNP of 177 pg/mL. The patient was intubated for acute hypoxemic respiratory failure. A chest X-ray revealed a large left-sided tension pneumothorax. Initial echocardiogram showed apical ballooning with a LVEF of 10–15%. A cardiac angiography revealed normal coronary arteries with no coronary disease. After supportive treatment, the patient's condition improved with a subsequent echocardiogram showing a LVEF of 60%. *Conclusion*. The patient was found to have Takotsubo cardiomyopathy in the setting of a tension pneumothorax. The exact mechanisms of ventricular dysfunction have not been clarified. However, multivessel coronary spasm or catecholamine cardiotoxicity has been suggested to have a causative role. We suggest that, in our patient, left ventricular dysfunction was induced by the latter mechanism related to the stress associated with acute pneumothorax.

## 1. Introduction

Takotsubo cardiomyopathy (TCM) is a unique transient form of cardiomyopathy frequently precipitated by a stressful event and has a clinical presentation similar to that of a myocardial infarction. This syndrome was first described by Sato et al. in 1990 [1] and has been increasingly reported in the literature. Various terminologies have been used, such as apical ballooning syndrome, stress-induced cardiomyopathy, and broken heart syndrome. However, Sharkey et al. [2] proposed the universal use of Takotsubo cardiomyopathy, which is now generally accepted.

The typical clinical features of TCM include chest pain and dyspnea [3, 4]. Postmenopausal women who recently went through a stressful event are most commonly affected [5]. Electrocardiographic findings may include transient ST-segment elevation and laboratory findings, a modest rise in cardiac troponin levels [3]. In the typical or most common variant, there is hypokinesis or akinesis of the mid and apical segments of the left ventricle and sparing of the basal ventricular segments seen on echocardiogram or left ventriculogram [3].

Uniquely, coronary angiography reveals no evidence of obstructive coronary disease or plaque rupture. Various criteria need to be met in order to make the diagnosis of TCM. They were recently revised and termed the Mayo Clinic Criteria [3, 4] (see* the Current Mayo Clinic Criteria for Takotsubo Cardiomyopathy*). Complications such as congestive heart failure have been reported. However the overall prognosis of TCM is good, as spontaneous recovery is found in most patients who are treated supportively.

*Current Mayo Clinic Criteria for Takotsubo Cardiomyopathy* [2, 3] are as follows.

1. Transient hypokinesia, akinesia, or dyskinesia in the left ventricular mid segments with or without apical involvement; regional wall motion abnormalities that usually extend beyond a single epicardial vascular distribution; and frequently, but not always, a stressful trigger.
2. The absence of obstructive coronary disease or angiographic evidence of acute plaque rupture. (*Some patients with TCM can exhibit some degree of coronary artery disease*.)
3. New ECG abnormalities (ST-segment elevations and/or T-wave inversion) or modest elevation in cardiac troponin.
4. Absence of pheochromocytoma and myocarditis.

The pathophysiology has not been clearly described. Catecholamine cardiotoxicity remains the most common explanation [3]. Broad arrays of clinical conditions have been reported as triggers of TCM and investigating these may improve our understanding of the pathophysiology. The triggers include severe emotional stress, physical stress including central nervous system pathology, severe illness, drug use/withdrawal, and severe pain [3, 4]. Here we report a case of TCM in the setting of a severely painful tension pneumothorax.

## 2. Case Report

**Figure 1:**
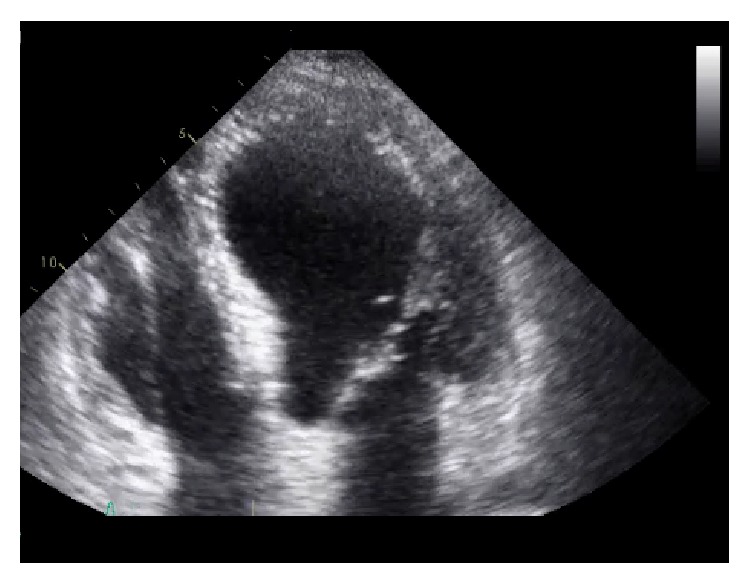
Echocardiogram with severely decreased EF of 13% and possible stress cardiomyopathy. PAP systolic 40 mmHg. Apical ballooning involving all left ventricular (LV) wall.

A 78-year-old female with a past medical history of diabetes mellitus type II, hypertension, osteoporosis, and asthma was brought into the emergency department by ambulance with severe shortness of breath. She was afebrile, tachypneic (respiratory rate of 28 rpm), with an elevated blood pressure of 185/105 mmHg and heart rate of 111 beats per minute. Her chest X-ray revealed a large left-sided tension pneumothorax. Because of impending respiratory failure, she was intubated and placed on mechanical ventilation. A chest tube was inserted with subsequent reexpansion of the left lung. The patient was admitted to the intensive care unit for monitoring. Cardiac biomarker, troponin I, was found to be elevated at 16.529 ng/mL (reference range: ≤0.1 ng/mL). Serial Electrocardiograms (ECGs) done over the course of 12 hours demonstrated sinus bradycardia, alternating with sinus tachycardia and normal sinus rhythm. Anterior Q waves were seen, along with dynamic T-wave changes and transient ST-segment elevation was also noted in the lateral leads. Transthoracic echocardiogram revealed global left ventricular hypokinesis sparing the basal segments, apical ballooning, and severe systolic dysfunction with an estimated left ventricular ejection fraction (LVEF) of 13% (Figure 1).

**Figure 2:**
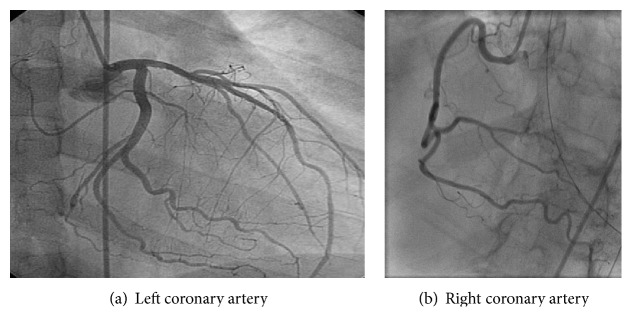
Cardiac catheterization revealing normal coronary arteries.

The patient was treated with aspirin, clopidogrel, a statin, and Ramipril and was subsequently transferred to another facility for cardiac catheterization. Coronary angiography revealed normal left and right coronary artery systems and no evidence of coronary artery disease (Figure 2). She was treated supportively and her condition improved over the next few weeks. A transthoracic echocardiogram performed 12 days later revealed no left ventricular dilation, resolved wall motion abnormality, and a significantly improved LVEF of 60%.

## 3. Discussion

The prevalence of TCM has been estimated to range from 1.7 to 2.2 percent of cases presenting with suspected acute coronary syndrome (ACS) [5–9]. TCM is much more common in elderly women with 80–100% of cases being female [10–13] and the mean age of presentation being 61 to 76 years [12]. Our patient fits these demographics, as she is an elderly postmenopausal female who was originally thought to be suffering from stress-induced acute coronary syndrome.

The clinical presentation of TCM is similar to that of an acute ST-segment elevation myocardial infarction (STEMI) [4, 6, 10, 14], with the most common presenting symptoms being acute substernal chest pain, dyspnea, nausea, and vomiting [4, 6]. We were unable to elicit these classic symptoms from our patient as she was sedated and intubated. The ECG and laboratory values were the clues that prompted cardiac concern.

The most common ECG finding in TCM is ST-segment elevation. Pawlak et al. compared the presentation between women who present with STEMI or TCM and found that the ST-segment was elevated in 70% of the TCM cases [14]. Other ECG findings included ST-segment depression (9.7%) and dynamic T-wave changes (17%) [14]. Other reported late ECG findings include development of diffuse T-wave inversion, prolongation of the QT interval, and rare, transient development of pathological Q waves [3, 10]. Our patient demonstrated the majority of these ECG changes, with transient ST-segment elevation, dynamic T-wave changes, pathological Q waves in leads I and aVL, and borderline QT prolongation.

Additionally, cardiac biomarkers are found to be modestly elevated in TCM. Pawlak et al. found that the median troponin I values for TCM patients are 2.99 ± 5.36 ng/mL compared to a median value of 42.70 ± 64.79 ng/mL in the STEMI patients [14]. Our patient had a peak troponin I value of 16.529 ng/mL, which is higher than the median value found in the literature, but significantly lower than the median value found in the patients having a STEMI. The Mayo Clinic Criteria describe a “modest” rise of cardiac biomarkers [3].

Echocardiography and left ventriculography demonstrate the characteristic regional wall motion abnormalities that define TCM. In the most common and classic variant there is hypokinesis or akinesis of the mid and apical segments of the left ventricle and sparing of the basal systolic function with the wall motion abnormality typically extending beyond the distribution of a single coronary artery [3]. LVEF is significantly reduced, mimicking an acute MI. In the study done by Pawlak et al., it was found that both patients suffering from a STEMI and TCM had a lower LVEF at initial presentation; however, follow-up echocardiograms demonstrated a significantly higher LVEF in the TCM patients than those who had STEMI [14]. Our patient's echocardiogram performed 12 days later demonstrated resolved wall motion abnormalities, no apical ballooning, and a normal LVEF of 60%, consistent with the transient nature of TCM.

## 4. Risk Factors and Pathogenesis

The unique feature of TCM is the occurrence of a preceding emotional or physical stressful event. In one study done at the Mayo Clinic, 98% of cases were found to have a preceding trigger [12, 15]. Documented emotional triggers are vast and include but are not limited to death or severe illness of a loved one, receiving bad news, divorce, relocation, and natural disasters [3]. Reported physical stressors are also very nonspecific and include surgery (cholecystectomy, hysterectomy), severe illness (asthma, chronic obstructive airway exacerbation, acute cholecystitis, pseudomembranous colitis, cocaine use, opiate withdrawal, and thyrotoxicosis), iatrogenic cases (dobutamine stress echo, exercise stress test), and severe pain (bone fracture, renal colic, pulmonary embolism, and pneumothorax) [3, 4]. Our patient presented in severe pain associated with a tension pneumothorax.

The exact mechanisms of transient left ventricular dysfunction have been disputed with the most commonly discussed possible one being catecholamine cardiotoxicity and the second most common being multivessel coronary spasm [16]. Other proposed factors include a genetic predisposition [17] or a protective role of estrogen as postmenopausal females are most likely at risk [18]. In the present patient, evidence of coronary vascular spasm was excluded by the negative coronary angiogram performed at the time of ST-segment elevation. As mentioned before, this patient is postmenopausal and family history of TCM was not elicited. It is our belief that the TCM in this patient was induced by an increase in catecholamines caused by the stress and pain resulting from tension pneumothorax. A review literature revealed that only one case of TCM triggered by a pneumothorax had been reported [19].

## 5. Diagnosis

The diagnosis of TCM should be considered in the differential diagnosis of a patient presenting with suspected acute coronary syndrome (ACS), especially postmenopausal females. The Mayo Clinic Criteria for the diagnosis of TCM (see* the Current Mayo Clinic Criteria for Takotsubo Cardiomyopathy*) are very helpful to evaluate and aid in management for patients with a suspicion of TCM [3]. Our patient met all four Mayo Clinic Criteria and was confirmed to have TCM.

Diagnosis is established with ECG and echocardiogram, with the latest one being the most practical imaging technique. Cardiac magnetic resonance (CMR), although not used in this case, is an emerging tool which can be used for differentiating TCM from myocardial infarction. CMR can evaluate possible ischemic damage and more accurately visualize the extent of ventricular dysfunction [3]. The only con is its limited availability and low tolerance in a number of patients making this study more like a complimentary imaging study able to provide additional information in a subset of patients with a nonclear clinical course [20].

## 6. Treatment and Prognosis

Although optimal management of TCM has not been established and spontaneous recovery of the cardiac function follows, it is important to treat the patient for possible ACS. At time of presentation, TCM will mimic ACS, and initial management should be directed towards management and support as if it is an acute myocardial infarction (AMI). Once the diagnosis of TCM is confirmed, with supportive care, cardiac recovery usually takes no longer than 4 to 8 weeks [3]. As mentioned previously it took our present patient less than 2 weeks to recover spontaneously with supportive therapy alone.

A number of complications have been seen in the acute phase of the disease and some studies have reported an in-hospital mortality of around 2%. Some recurrence has been documented also [19].

## 7. Conclusion

Takotsubo cardiomyopathy is a distinct reversible cardiomyopathy resulting from the effects of stress. Since it generally mimics ACS it should be included in the differential diagnosis of ACS, especially if the patient is a postmenopausal female. It is important to identify the presence of emotional or physical stressors, including conditions that can cause severe pain such as tension pneumothorax in the case described. This diagnosis can only be made after proof of recovery has been obtained, stressing the need to carefully explain to our patients the importance of clinical follow-up.
